# Comparison of L-Threonine Aldolase Variants in the Aldol and Retro-Aldol Reactions

**DOI:** 10.3389/fbioe.2019.00119

**Published:** 2019-05-28

**Authors:** Kateryna Fesko

**Affiliations:** Institute of Organic Chemistry, Graz University of Technology, Graz, Austria

**Keywords:** biocatalysis, asymmetric synthesis, amino acids, threonine aldolase, aldol reaction

## Abstract

Most of biochemical and mutagenesis studies performed with L-threonine aldolases were done with respect to natural activity, the cleavage of L-threonine and sometimes L-β-phenylserine. However, the properties of variants and the impact of mutations on the product synthesis are more interesting from an applications point of view. Here we performed site-directed mutagenesis of active site residues of L-threonine aldolase from *Aeromonas jandaei* to analyze their impact on the retro-aldol activity and on the aldol synthesis of L-β-phenylserine and L-α-alkyl-β-phenylserines. Consequently, reduced retro-aldol activity upon mutation of catalytically important residues led to increased conversions and diastereoselectivities in the synthetic direction. Thus, L-β-phenylserine can be produced with conversions up to 60% and *d.e*.‘s up to 80% (*syn*) under kinetic control. Furthermorem, the donor specificity of L-threonine aldolase was increased upon mutation of active site residues, which enlarged the pocket size for an efficient binding and stabilization of donor molecules in the active site. This study broadens the knowledge about L-threonine aldolase catalyzed reactions and improves the synthetic protocols for the biocatalytic asymmetric synthesis of unnatural amino acids.

## Introduction

**Figure 1:**
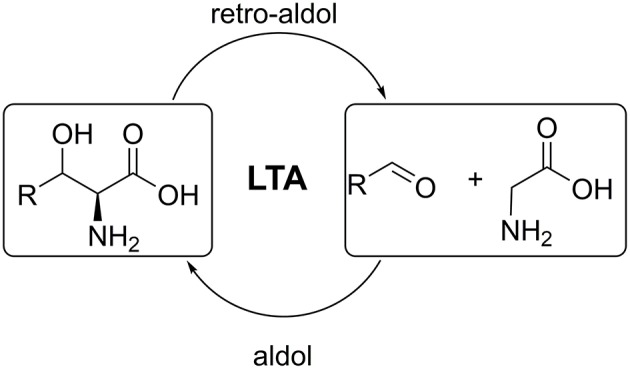
Retro-aldol and aldol reactions catalyzed by L-threonine aldolases.

L-threonine aldolases (LTAs) catalyse the reversible cleavage of L-threonine and non-natural L-β-hydroxy-α-amino acids to produce glycine and the corresponding aldehydes. The aldol addition reactions catalyzed by LTAs have great biotechnological potential for asymmetric carbon-carbon bond formation toward amino acids (Figure 1) (Fesko and Gruber-Khadjawi, 2013). The products of such reaction, β-hydroxy-α-amino acids, are important building blocks for many complex natural products and pharmaceuticals (Breuer et al., 2004; Baltz et al., 2005; Panke and Wubbolts, 2005; Goldstein, 2006). LTAs show high stereospecificity at the α-carbon, thus strictly only L-products are formed. This makes LTA a promising candidate for the stereoselective asymmetric synthesis of unnatural L-amino acids (Dückers et al., 2010; Franz and Stewart, 2014). LTAs are ubiquitous in nature and were found in bacteria, fungi and in mammalian species. Around 5,000 sequences annotated as LTA can be currently found in databases (e.g., NCBI, Swiss-Prot). However, only several genes have been subjected to structural, mechanistic, and biochemical studies (Fesko, 2016). These studies allowed scientists to elucidate key functional residues in LTAs and draw general conclusions on the mechanism of catalysis involved (Kielkopf and Burley, 2002; di Salvo et al., 2014; Qin et al., 2014).

**Figure 2:**
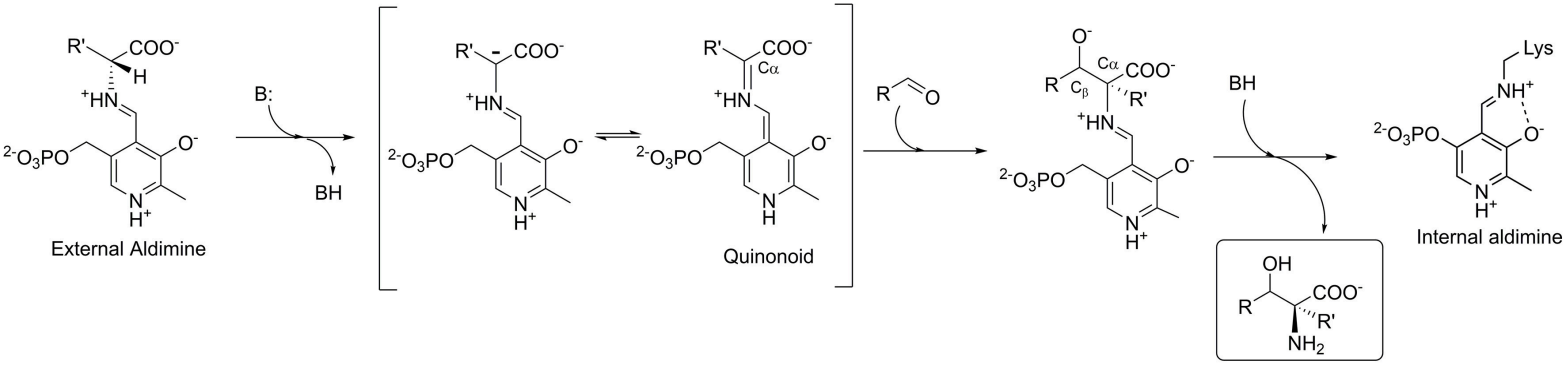
Mechanism of the aldol addition to synthesize L-β-hydroxy-α-amino acids catalyzed by L-threonine aldolase.

LTAs require pyridoxal-5′-phosphate (PLP) as cofactor, which is covalently bonded to the apo-enzyme via the active site lysine residue. In the aldol addition reaction, an amino acid donor substrate substitutes lysine to form an external aldimine with PLP. The C_α_-proton of the donor is then abstracted to form a carbanion, which is stabilized by delocalization in the aromatic moiety of the PLP-ring through formation of a quinonoid complex (Figure 2). The cofactor's ability to stabilize a carbanion is determined by a complex network of the PLP-surrounding residues (Fesko et al., 2018). Next, an aldehyde substrate enters from the *re*-side of the PLP ring to form a C_α_-C_β_ bond via an aldol addition mechanism. Subsequent to the protonation of the C_β_-oxygen, an L-β-hydroxy-α-amino acid product is released (Figure 2). The stereoselectivity at the C_α_ of the product is stringently controlled by the enzyme, whereas the selectivity at the C_β_ bearing the hydroxyl group is often moderate and depends on the nature of substrate used (Dückers et al., 2010).

In spite of the promising opportunities to use LTAs in the biocatalytic production of non-natural amino acids, their broad application has been hindered by often moderate yields and low diastereoselectivities obtained in the aldol reaction. Thus, several directed evolution studies have been conducted to improve the stability, substrate specificity and stereoselectivity of LTAs (Franz and Stewart, 2014; Fesko, 2016). Interestingly, none of these attempts led to a variant with a significantly increased catalytic activity and diastereoselectivity. Thus, reaction engineering or rational design of enzymes, ideally in combination, might be a useful strategy for the improvement of LTA-catalyzed reactions. As suggested before, rapid epimerization of *syn*- and *anti*-isomers, leading to the fast equilibration and thus low diastereoselectivity, can be governed by appropriate protein engineering focusing on the most relevant catalytic reaction steps (Fesko et al., 2008; Franz and Stewart, 2014). Thus, understanding the detailed mechanistic features and role of the active site residue is of paramount importance for the successful rational design of LTAs.

To find catalytically important residues, a multiple sequence alignment of LTA sequences was performed recently (Giger et al., 2012). Further, the residues, which are important for the reaction specificity were identified by structure-sequence comparison of the enzymes belonging to the fold type I of PLP-dependent enzymes with different reaction specificities (Fesko et al., 2018). These studies allowed identifying catalytic core residues conserved within LTAs. Interestingly, the functional characterization of the mutants produced in previous mutagenesis studies, as well as the screening of the LTAs variants derived from directed evolution was mainly done in the retro-aldol direction with the natural substrate L-threonine (Giger et al., 2012; Bulut et al., 2015). Clearly, in view of the applications, the effect of mutations on the aldol addition to synthesize structurally different β-hydroxy-α-amino acids is far more relevant. In our current study, we have used L-threonine aldolase from *Aeromonas jandaei* (LTAaj) with known broad donor specificity (Fesko et al., 2010), to evaluate the impact of mutations of active site residues on the catalytic efficiency in the aldol addition direction, the diastereoselectivity, and the donor specificity. The variants were constructed, recombinantly overexpressed, purified to homogeneity, and tested as catalysts for the aldol addition of glycine, D-alanine, or D-serine to benzaldehyde as the model reaction. The contribution of mutations on the conversion in the aldol addition reaction is compared with their impact on the activity in the retro-aldol reaction.

## Materials and Methods

### Chemicals and Solvents

All chemicals were purchased from Sigma Aldrich, unless stated otherwise. All enzymes for the genetic work were purchased from ThermoFisher Scientific. The expression vector pEamTA was produced as stated before (Reisinger et al., 2007). The *Escherichia coli* strain BL21(DE3) was obtained from BioLabs (New England). Analytical HPLC was carried out with an Agilent 1,200 HPLC-MS system equipped with a 6,120 quadrupol mass spectrometer. UV-vis scans and single wavelength kinetics were measured on a Perkin Elmer Lambda 2 spectrophotometer.

### Cloning of LTAaj and Site-Directed Mutagenesis

The wild-type LTAaj (for sequence and alignment with PDB:3WGB template see Supplementary Information) was cloned in pEamTA vector with a C-terminal His-tag as previously described (Fesko et al., 2015). LTAaj mutants were generated by site-directed mutagenesis according to Agilent's QuikChange II protocol using PfuUltra High-Fidelity DNA Polymerase (Agilent Technologies). The pEamTA-LTAaj plasmid was used as the template. Primers were ordered at Integrated DNA Technologies. PCR was carried out under the following conditions: 50 μL total volume, 200 μM of each dNTP, 0.2 μM of each primer, 5 ng of template plasmid DNA, and 2.5 U of PfuUltra High-Fidelity DNA polymerase. Cycling conditions were as follows: initial denaturation at 95°C for 2 min, followed by 21 cycles of denaturation at 95°C for 30 s, annealing at 55°C for 60 s, extension at 68°C for 8 min. Amplification was controlled by electrophoresis of 10 μL of the PCR reaction on an agarose gel. After the digestion of parental non-mutated methylated plasmid DNA with DpnI at 37°C for 1 h, the samples were desalted and electro-competent *Escherichia coli* BL21(DE3) cells were transformed with the resulting nicked vector DNA containing the desired mutations. The introduction of mutations was confirmed by sequencing analysis (LGC genomics).

### Protein Expression and Purification

The constructs pEamTA-LTAaj-mut contained the *tac* promoter and the LacI repressor, which control the protein expression level. 300 mL 2xYT medium (in a 2 L flask) containing ampicillin (50 μg/mL) was inoculated with 5 mL of an *E. coli* overnight culture. Cells were grown at 37°C to an OD_600_ of 0.6–0.8. Protein expression was induced by the addition of 1 mM IPTG and the culture was allowed to grow overnight at 20°C. The biomass of the bacterial cultures was harvested by centrifugation at 4500x g for 10 min at 4°C. The cell pellets were re-suspended in sodium phosphate buffer (0.1 M, pH 7.4) and disrupted by ultrasonic treatment for 7 min. The crude lysate was cleared by centrifugation at 20,000 x g for 1 h at 4°C and subjected to further purification. Lysates were loaded onto a Ni-NTA Superflow resin (Qiagen), washed with sodium phosphate buffer (50 mM, pH 7.4) containing imidazole (5 mM) and NaCl (0.3 M) and subsequently eluted with imidazole (0.3 M) in sodium phosphate buffer (50 mM, pH 7.4). To remove the imidazole, the pooled and concentrated protein fractions were loaded on a Sephadex desalting column PD-10 (GE Healthcare) and eluted with sodium phosphate buffer (50 mM, pH 7.4) containing 0.1 mM PLP. The purified proteins were used for the activity measurements and biocatalytic transformations.

### Enzyme Activity Assay

The activity of the LTAaj mutants was verified by monitoring the formation of benzaldehyde upon cleavage of DL-*syn*-β-phenylserine (30 mM) or L-α-methyl-β-phenylserine (55:45 *syn*:*anti* mixture; 30 mM) at 279 nm (ε = 1.4 × 10^3^ M^−1^ cm^−1^). The kinetic assays were carried out at 25°C in sodium phosphate buffer (50 mM, pH 8) containing PLP (50 μM) and LTA (10–50 μM). One activity unit (U) is defined as the amount of enzyme that catalyzes the formation of 1 μmol of benzaldehyde per minute under these conditions. Controls without the addition of TAs were carried out under the same conditions.

### Spectroscopic Analysis of LTAaj Variants

The spectral scanning of purified LTAaj and mutants was performed in 1 mL sodium phosphate buffer (50 mM, pH 8.0) with LTA (50 μM) in a 1 cm light path cuvette at the wavelength range of 300–550 nm at 22°C. The formation of an external aldimine (λ = 420 nm) and quinonoid intermediate (λ = 495 nm) was monitored after addition of a donor substrate (0.1 M final concentration).

### Chromatographic Analyses

For substrate and product quantification by HPLC, reference DL-*syn*-β-phenylserine and L-α-methyl-β-phenylserine were measured in different concentrations (0.05–2.5 mM) to calculate standard curves for each compound. Concentrations were calculated using linear interpolation.

The HPLC-UV with pre-column derivatization was performed on an Agilent 1,200 Series HPLC system for *e.e*. and *d.e*. determination. The reaction was first quenched by diluting 1:50 with NTBB buffer (0.1 M of Na_2_B_4_O_7_, pH 10.5), and the samples were treated with ortho-phthaldialdehyde/N-acetyl-L-cysteine (OPA/NAC). The OPA/NAC reagent was obtained by dissolving of 15 mg of NAC in 300 μL of distilled water and 300 μL of NTBB, and then 10 mg of OPA in 400 μL of MeOH was added. A freshly prepared solution was always used. 5 μL of OPA/NAC reagent was mixed with 1 μL of diluted reaction solution for 5 or 15 min (for α-quaternary products) in the HPLC system before injection. The molar ratio of OPA and the total of amino acids was maintained at 3.5: 1. Detection of the analytes was accomplished with a diode array detector at a wavelength of λ = 340 nm. The separations were carried out on an EC 150/3 Nucleodur® C18 Gravity column (150 × 3.0 mm, 3.0 μm; Macherey-Nagel, Düren, Germany). The mobile phases were 85% potassium phosphate buffer (20 mM, pH 6.8) and 15% acetonitrile (ACN) isocratic flow at 2.5 mL/min.

For HPLC-MS general monitoring of the reactions, eluent mixtures consisting of methanol, and water containing 0.01 vol% formic acid as additive were used. UV (210 nm) and ESI-MS detection, typically in positive mode, was used to identify the compounds. Following settings of the Agilent 6,120 MSD were applied: drying gas: N_2_, 10.5 L/min; drying gas temperature: 350°C; nebulizer pressure: 35 psi; capillary voltage: 3,000 V; mode: negative; scan parameters: mass range: 100–450, fragmentor: 30 V, gain: 1.0, threshold: 150, step size: 0.10. The separation of the analytes was carried out using a Poroshell® 120 EC-C18, 3.0 × 100 mm, 2.7 μm (Agilent, Vienna, Austria) reversed-phase column. Samples were either dissolved in methanol and any insoluble particles were removed prior to analysis by centrifugation or filtration through 0.2 μm GHP Acrodic® 13 mm syringe filters (PN 4,567, Pall Corporation, Ann Arbor, USA). A stepwise gradient was used: 0.0 min: 98 % water/ 0.1% HCOOH and 2% CH_3_OH, linear gradient to 100% CH_3_OH in 6 min, 6.0–8.0 min 100% CH_3_OH; 0.7 mL/min, 30°C.

### General Procedure for the Synthesis of L-β-Phenylserine 3a

The enzymatic transformations were performed in 1 mL reaction volume in sodium phosphate buffer (50 mM, pH 8.0), containing PLP (50 μM), DMSO (10%, v/v), benzaldehyde (50 mM) and glycine (0.5 M). Transformations were performed at 30°C with 0.5 U (based on phenylserine cleavage activity) of protein. To stop the reaction, 50 μL of the reaction mixture was diluted in 950 μL sodium tetraborate buffer (0.1 M, pH 10.5) and used for the determination of conversion by RP-HPLC after a pre-column derivatization with OPA/NAC.

*L-*β*-phenylserine* **3a**: t_L−syn_ = 4.2 min, t_L−anti_ = 7.5 min.

*L-*β*-(3-nitro)-phenylserine* **4a**: t_L−syn_ = 6.7 min, t_L−anti_ = 10.1 min.

### General Procedure for the Synthesis of L-α-Alkyl-β-Phenylserine 3b-d, 4b-g

The enzymatic transformations were performed in 1 mL reaction volume in sodium phosphate buffer (50 mM, pH 8.0), containing PLP (50 μM), DMSO (10%, v/v), 50 mM of benzaldehyde or 3-nitro-benzaldehyde and 0.5 M of D-amino acid donor (D-alanine, D-serine, D-aminobutanoic acid, D-valine, D-norvaline). Transformations were performed at 25°C with 10 U (based on phenylserine cleavage activity) of protein. To stop the reaction, 50 μL of the reaction mixture was diluted in 450 μL of 50/50 methanol/formic acid 0.01% and used for the HPLC-MS analysis.

*L-*α*-methyl-*β*-phenylserine* **3b**: t_L−anti_ = 2.7 min, t_L−syn_ = 4.1 min; [M+1]^+^ 195.9

*L-*α*-hydroxymethyl-*β*-phenylserine* **3c**: t_L−anti_ = 1.3 min, t_L−syn_ = 1.8 min; [M+1]^+^ 212.1

*L-*α*-ethyl-*β*-phenylserine* **3d**: t_L−anti_ = 2.1 min, t_L−syn_ = 2.3 min; [M+1]^+^ 210.1

*L-*α*-methyl-*β*-(3-nitro)-phenylserine* **4b**: t_L−anti_ = 3.1 min, t_L−syn_ = 4.4 min; [M+1]^+^ 241.2

*L-*α*-hydroxymethyl-*β*-(3-nitro)-phenylserine* **4c**: t_L−anti_ = 1.6 min, t_L−syn_ = 2.1 min; [M+1]^+^ 257.2

*L-*α*-ethyl-*β*-(3-nitro)-phenylserine* **4d**: t_L−anti_ = 2.3 min, t_L−syn_ = 2.5 min; [M+1]^+^ 255.0

*L-*α*-propyl-*β*-(3-nitro)-phenylserine* **4f**: t_L−anti_ = 2.6 min, t_L−syn_ = 2.8 min; [M+1]^+^ 269.2

*L-*α*-(2-hydroxyethyl)-*β*-(3-nitro)-phenylserine* **4g**: t_L−anti_ = 2.1 min, t_L−syn_ = 2.2 min; [M+1]^+^ 271.2

## Results

**Figure 3:**
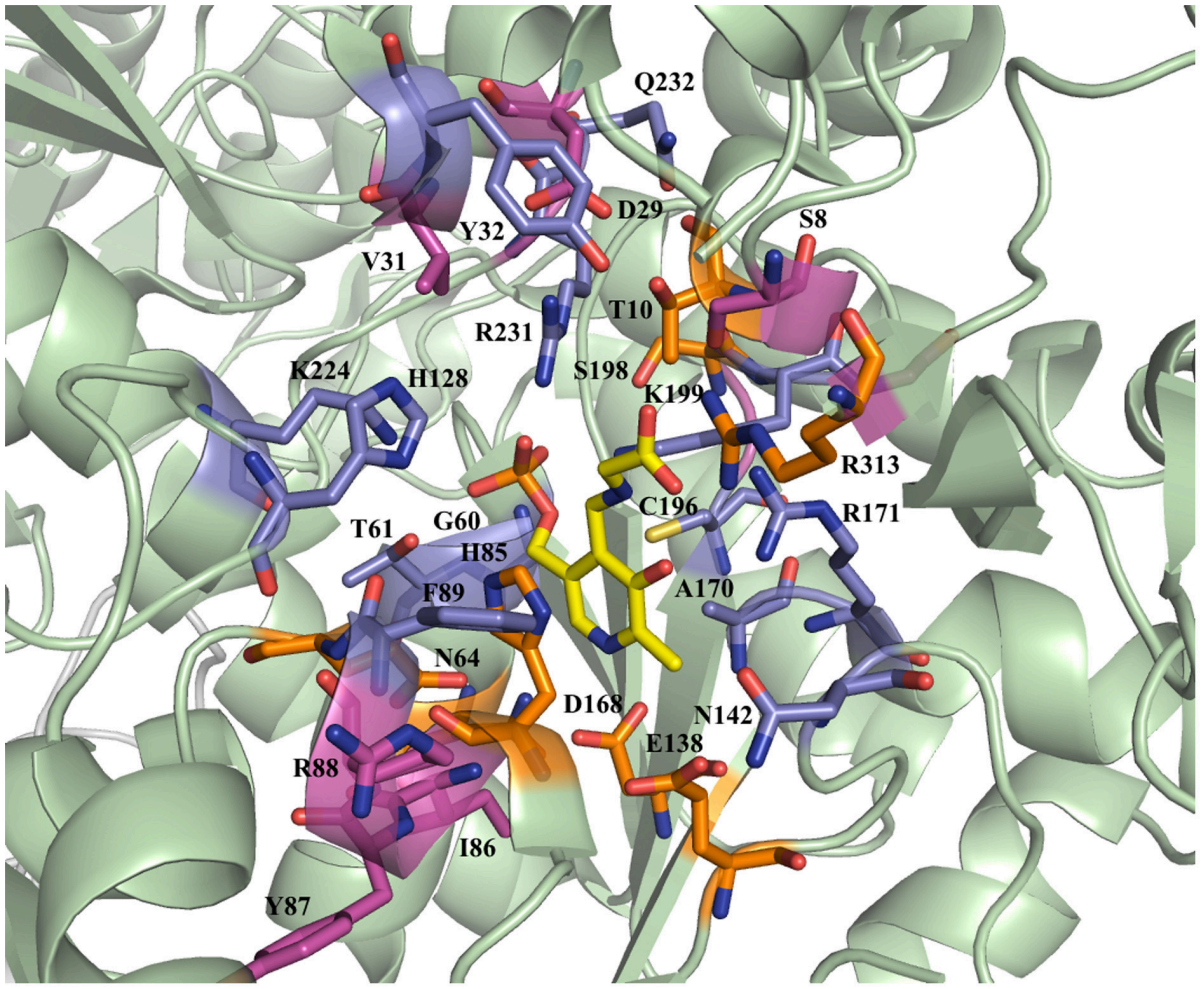
Active site residues of LTAaj (PDB: 3WGB) tackled by site-directed mutagenesis in this study (Yellow—PLP-glycine external aldimine; orange—mutations with a positive effect on conversion in the aldol synthesis of L-β-phenylserine; lila—with negative effect; pink—no effect).

**Figure 4:**
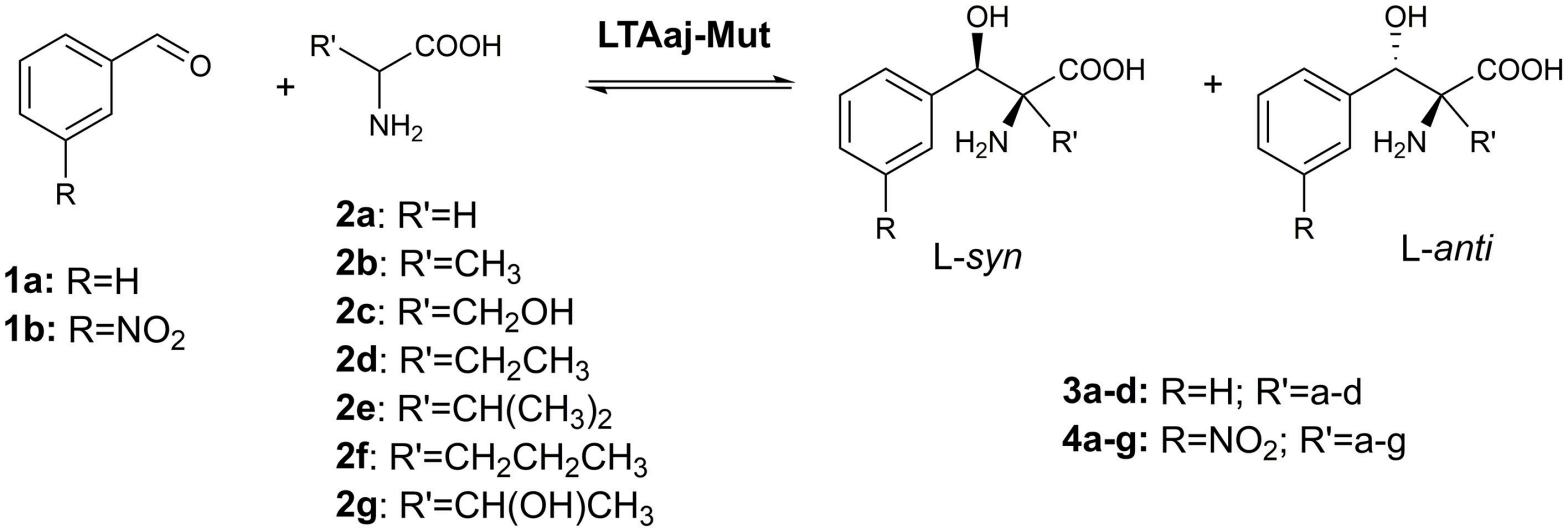
Aldol addition of aldehyde **1** to an amino acid donor **2** catalyzed by the LTAaj variants.

The active site residues, which are located within 5Å from PLP-Glycine complex in LTAaj (reference PDB: 3WGB), were selected for the site-directed mutagenesis to test their influence on the outcome in aldol reactions (Figure 3). LTAs accept a wide range of aliphatic and aromatic aldehydes as an electrophilic substrate, whereas glycine is accepted as the only nucleophile donor by most of the known LTAs (Steinreiber et al., 2007a,b; Franz and Stewart, 2014). LTAaj is a unique enzyme with broad donor specificity, accepting other small-sized D-amino acids as donors (Fesko et al., 2010; Blesl et al., 2018). The aldol additions of benzaldehyde (**1a**) to amino acid donors [glycine (**2a**), D-alanine (**2b**), D-serine (**2c**), and D-aminobutanoic acid (**2d**)] to produce L-β-phenylserine (**3a**) and L-α-alkyl-β-phenylserine **3b-d**, respectively, were used as model reactions in this study to compare the properties of the LTAaj mutant variants (Figure 4). The best mutants were also tested with 3-nitro-benzaldehyde (**1b**) substrate as evidence that obtained features can be extended to reactions with other aldehydes.

### Favorable Mutations for the Synthesis of L-β-Phenylserine (3a)

**Figure 5:**
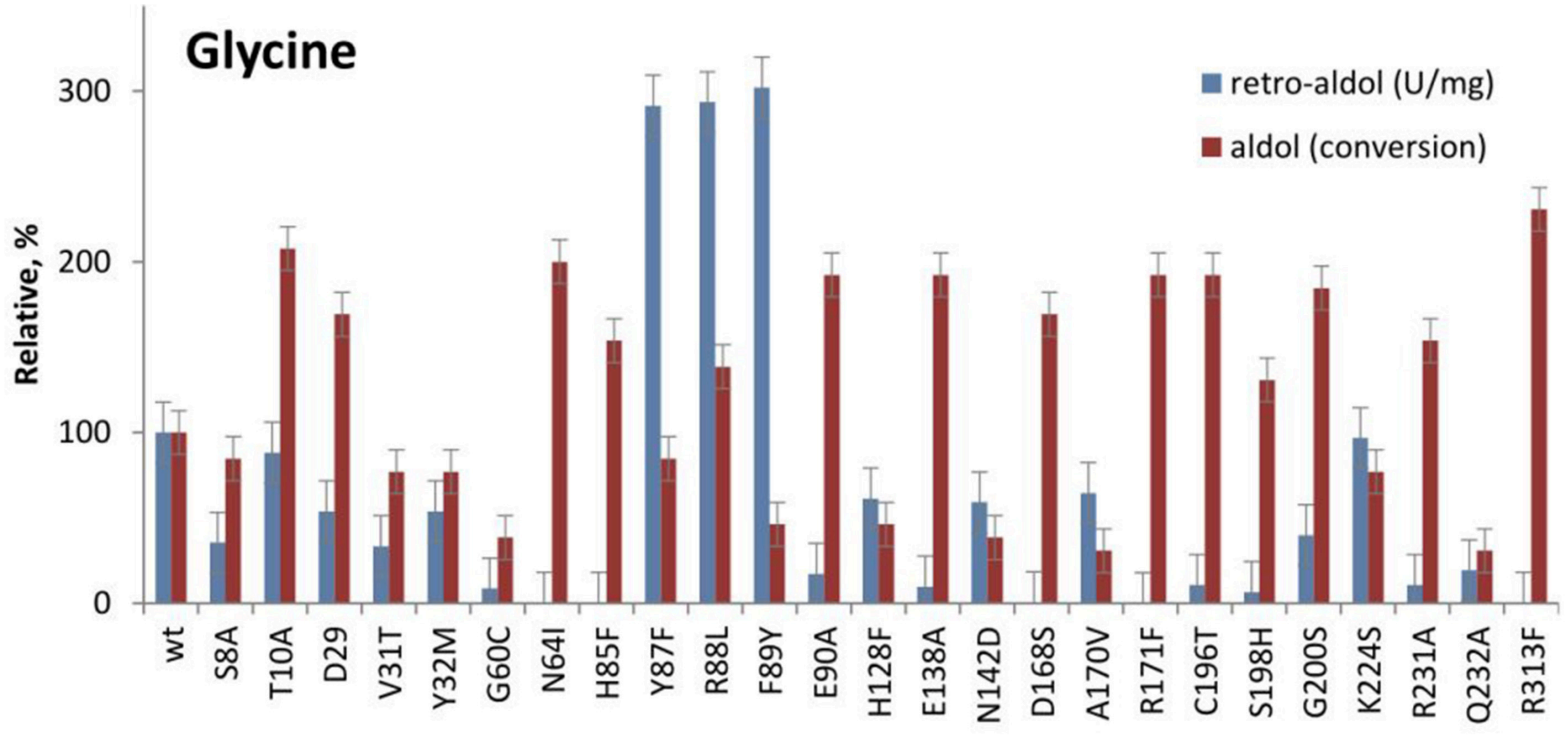
Relative catalytic activity in the retro-aldol cleavage and conversion after 24 h in the aldol synthesis of L-β-phenylserine catalyzed by LTAaj mutants.

According to the phylogenetic analysis of LTA sequences, LTAs can be grouped into two major functional families—L-*allo*-TA and L-low specificity TA, which correlates with their specificity at C_β_ of L-threonine (Liu et al., 2015; Fesko et al., 2018). The sequence similarity is low between functional families and only a few conserved residues were found in all LTA sequences (G60, T61, N64, H85, E90, E138, D168, R171, and R313 in LTAaj). It is not surprising, that their mutation led to a significant loss of activity in the cleavage of L-threonine and L-β-phenylserine (Fesko et al., 2018). In this study, we have tested the performance of these and other active site mutants in the aldol addition reactions. Interestingly, the variants N64I, H85F, E90A, E138A, D168S, C196T, G200S, R231A, R313F with a significantly decreased retro-aldol activity catalyse the formation of **3a** with conversions similar or even higher than the wild type enzyme although with slower rates (Figure 5, Table 1). For example, the R313F mutant with 500 fold reduced catalytic activity in the cleavage reaction, produces **3a** with 40% conversion after 24 h and 70% after 5 days.

**Table 1:** The catalytic activity of the LTAaj mutants in the retro-aldol cleavage reaction of L-β-*syn*-phenylserine (**3a**) and L-α-methyl-β-phenylserine (**3b**).

**Position**	**Mutants**	**Retro-aldol**		**Aldol^a^**							
		**3a**	**3b**	**2a**		**2b**		**2c**		**2d**	
		**U/mg**	**U/mg**	**Conv.%**	**d.r**.	**Conv.%**	**d.r**.	**Conv.%**	**d.r**.	**Conv.%**	**d.r**.
wt	–	93	1.1	38	72:28	0	–	0	–	0	–
				15^b^	60:40	23 (52)^b^	65:35	8^2^	45:55	0	–
S8	A	32.6	n.d.	11	87:13	18	55:45	8	48:52	0	–
T10	A	81.8	1.7	27 (55)^b^	67:33	31 (73)	75:35	10	45:55	0	–
	D	130.3	n.d.	5	63:38	22	55:45	9	47:53	0	–
D29	A	49.7	6.2	22	67:33	21	55:45	4	50:50	4	n.d.
V31	Y	30.8	1.3	10	52:48	20	60:40	8	50:50	n.d.	–
	R	12.1	n.d.	12	70:30	23	60:40	13	48:52	n.d.	–
	T	14.1	1.8	4	70:30	30	55:45	15	45:55	4	68:32
Y32	M	49.4	1.3	10	70:30	20	50:50	10	37:63	< 1	n.d.
	H	8.96	0.9	8	64:36	25	63:37	9	50:50	< 1	n.d.
	G	6.9	n.d	5	60:40	17	55:45	6	45:55	0	–
G60	C	8.3	0.9	5	60:40	13	60:40	6	47:53	0	–
N64	I	0.22	n.d.	39 (58)	63:37	0	–	0	–	0	–
H85	F	0.2	0.05	11 (21)	15:85	0	–	0	–	0	–
	Y	0.02	n.d.	17	55:45	0	–	0	–	0	–
Y87	D	271	1.1	11	75:25	30	50:50	9	45:55	< 1	n.d.
R88	L	273	1.6	18	70:30	23	60:40	8	45:55	< 1	n.d.
F89	Y	281	n.d.	6	70:30	15	45:55	9	48:52	< 1	n.d.
E90	A	16	n.d.	25	65:35	4	70:30	2	60:40	0	–
H128	F	57	n.d.	6	65:35	27	55:45	9	44:56	< 1	n.d.
	Y	102	2.3	5	70:30	35	60:40	14	46:54	< 1	n.d.
E138	A	8.8	0.2	25	65:35	3	85:15	1	65:35	< 1	n.d.
N142	D	54.8	n.d.	5	70:30	31	60:40	6	60:40	0	–
D168	S	0.54	0	22	60:40	0	–	0	–	0	–
A170	V	60.3	n.d.	4	70:30	11	48:52	8	45:55	0	–
R171	F	0.13	0.05	5	30:70	0	–	0	–	0	–
C196	T	9.9	n.d.	25	60:40	20	55:45	3	45:55	0	–
S198	H	6.3	n.d.	17	65:35	16	65:35	4	60:40	0	–
G200	S	37.2	n.d.	24	65:35	23	60:40	9	45:55	< 1	n.d.
K224	S	89.5	n.d	10	80:20	30	65:35	10	50:50	< 1	n.d.
R231	A	9.7	4.5	20	70:30	14	60:40	9	45:55	< 1	n.d.
Q232	A	18	n.d	4	60:40	13	58:42	6	50:50	4	67:33
R313	F	0.2	0	30 (61)	55:45	0	–	0	–	0	–
H85F/H128F		4.5	0	20	75:25	2	50:50	0	–	0	–
H85F/H128Y		4.5	0	5	60:40	2	40:60	0	–	0	–
R231A/G200S		23	n.d	11	60:40	20	65:35	5	60:40	< 1	20:80
R231A/Y32M		27	11.1	20	80:20	17	60:40	7	50:50	8	45:55
R231A/Y32G		6.5	n.d	22	80:20	12	55:45	1	50:50	2	50:50
R231A/H85F		10	0	19	80:20	1	37:63	0	–	0	–
R231A/S198H		7	n.d	20	80:20	22	60:40	10	55:45	2	25:75
R231A/V31R		27	2.1	5	60:40	18	55:45	3	60:40	n.d.	–
R231G/T10G		5.1	n.d	11	75:25	18	60:40	3	55:45	n.d.	–

*Conversion and the diastereomeric ratio (syn:anti) after 24 h in the aldol addition of benzaldehyde (**1a**) to glycine (**2a**), D-alanine (**2b**), D-serine (**2c**), and D-aminobutanoic acid (**2d**) catalyzed by LTAaj mutants*.

a *Reaction conditions: 0.5 U (for **2a**) or 5U (for **2b-d**) of enzyme, 50 mM of **1a**, 500 mM of **2a-c**, 20 mM NaH_2_PO_4_ buffer pH 8.0, 1 mL, 25°C. Products detected by HPLC/LC-MS; e.e._L_>99%*.

b *10U of wt LTAaj*.

c *in brackets reaction time 5 days*.

*n.d., not determined*.

**Figure 6:**
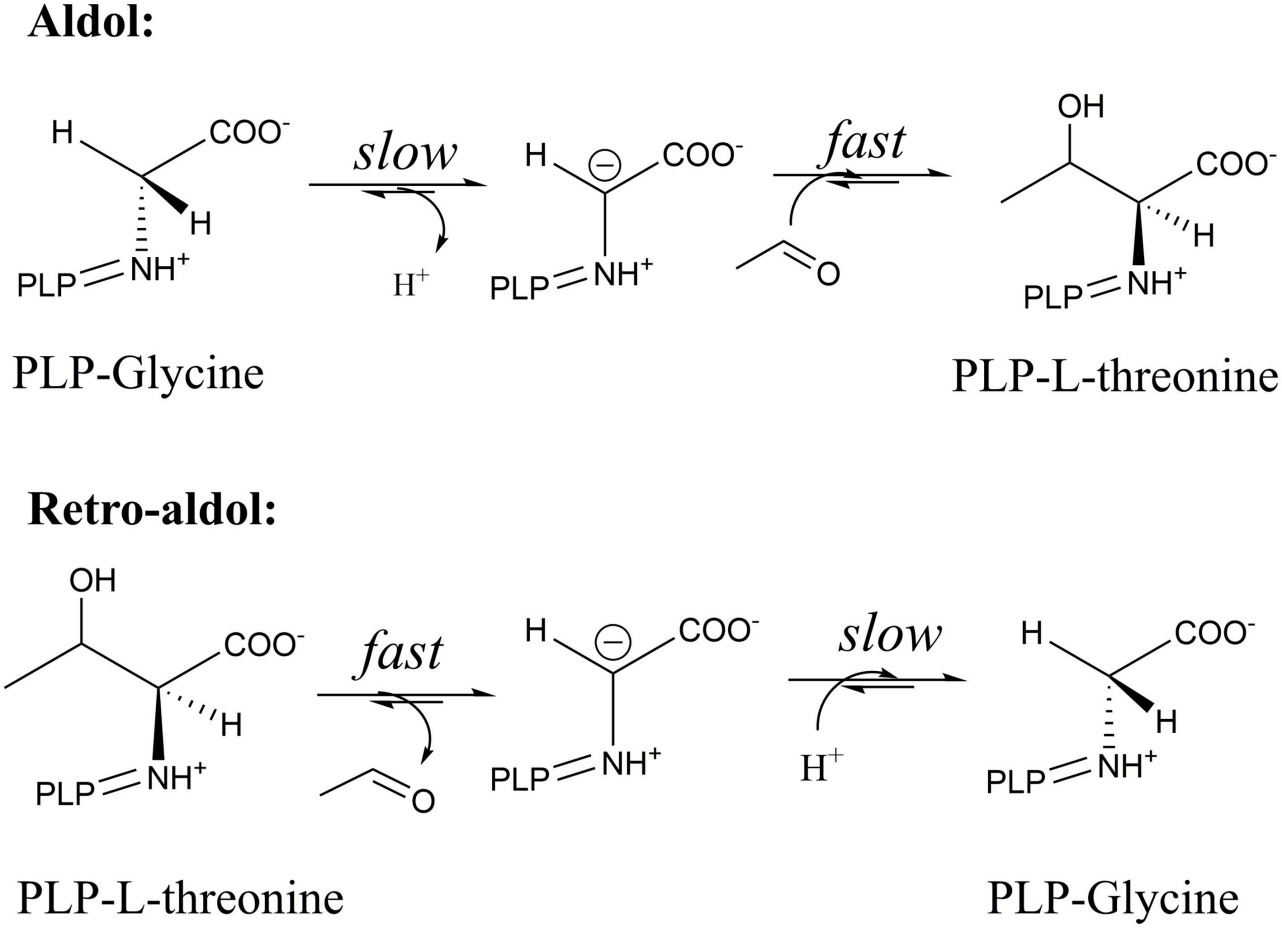
Relative rates of subsequent reaction steps in the aldol and retro-aldol reaction catalyzed by LTAaj.

As was shown before, these conserved residues are essential to control the electrophilic strength of the PLP cofactor to act as an electron sink stabilizing the formed carbanion after C_α_-C_β_ bond cleavage by the formation of quinonoid species of the PLP-glycine complex (Figure 2) (Fesko et al., 2018). This seems to be important for the retro-aldol reaction, but less essential in the opposite reaction—formation of the β-hydroxy-α-amino acid. Previously, we have shown, that the rate-limiting step in the LTA catalyzed reactions is the C_α_-H deprotonation of glycine donor (aldol direction) or protonation of the C_α_ carbanion to form glycine (retro-aldol direction) (Figure 6) (Fesko et al., 2008). Thus, in the retro-aldol reaction, the life-time of the glycine carbanion is longer in comparison to the aldol reaction and has to be stabilized via delocalization of electron density in the PLP-ring.

**Figure 7:**
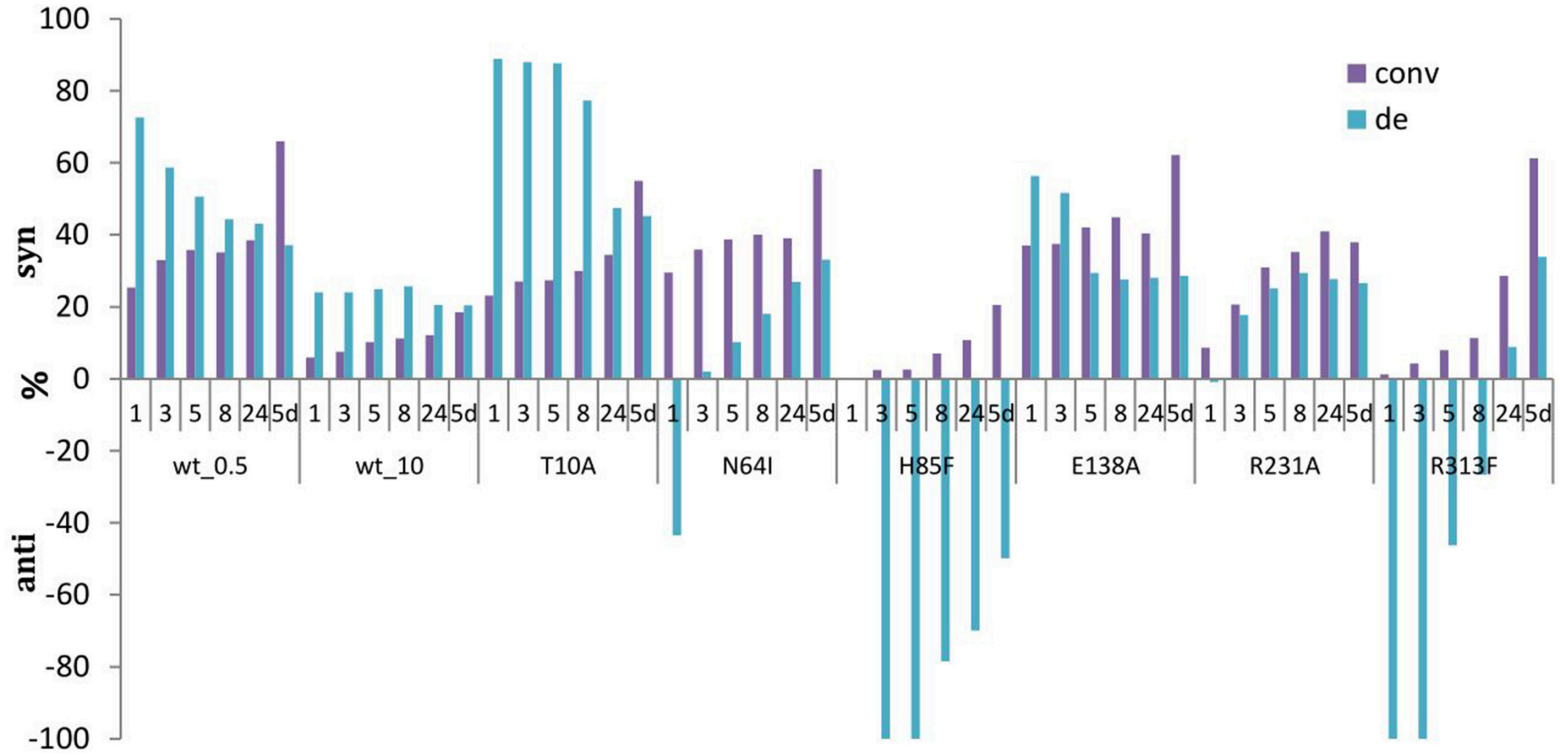
Development of conversions and diastereoselectivities within reaction times (1 h-5 d) in the aldol synthesis of L-β-phenylserine catalyzed by LTAaj mutants. (0.5 U per 1 mL reaction of LTAaj variants and wt_0.5, 10 U per 1 ml reaction of wt_10 were used in the reaction; *d.e*. = ((*syn*-*anti*)/(*syn*+*anti*)^*^100%).

The significantly higher energy barrier for the C_α_-H protonation step in comparison to the C_α_-C_β_ bond cleavage step requires a more stable carbanion in the case of retro-aldol cleavage (Fesko et al., 2008). The above described catalytic residues influence carbanion stability and thus suppress the retro-aldol reaction. Due to inhibition of reverse step, in the aldol addition reaction the equilibrium is reached later leading to the accumulation of the aldol products, when an excess of a donor is applied. Similar behavior was observed earlier for D-threonine aldolases, when aldol reactions were performed at low temperatures to suppress the cleavage reaction and to obtain aldol products under kinetic control (Steinreiber et al., 2007b). However, this was not possible to achieve in LTA catalyzed reactions due to its very high retro-aldol activity and fast equilibration even at low temperatures (Gutierrez et al., 2008). Here we observed mutations with intrinsically low retro-aldol activity, what allowed running the aldol reaction under kinetic control. Thus, conversion up to 60% was obtained for LTAaj variants with **1a** and **2a**, whereas only 20% was previously reported for the wild type enzyme under standard conditions (Figure 7, Table 1) (Blesl et al., 2018).

Moreover, in previous studies high excess of LTA was usually applied for the biocatalytic aldol reaction (5–10 U in a 1 mL reaction according to different studies) (Steinreiber et al., 2007a,b; Gutierrez et al., 2008; Dückers et al., 2010). This led to faster equilibration and the thermodynamically-controlled mixtures of products (*syn*/*anti*) was obtained. In the case of “low active” mutants or reducing the amount of wild type enzyme in the reaction mixture to 0.5 U, the products can be obtained under kinetically controlled conditions, when *syn*-isomer (in case of L-β-phenylserine) is formed faster than *anti*-isomer, leading to *d.e*. up to 80% and conversion of 20% after 5 h of reaction time (e.g., T10A mutant, Figure 7). Interestingly, some mutations cause the inversion of stereopreferences in the kinetically-controlled mode, thus *anti*-product was obtained as the major isomer for N64I, H85F, and R313F mutants. To conclude, for an efficient retro-aldol cleavage process catalyzed by LTA, the C_α_-carbanion intermediate has to be stabilized, whereas in the aldol process this is much less important. Thus, lowering the retro-aldol activity opens the possibility to run the aldol reaction under kinetic control to reach high diastereoselectivities with good conversion.

### Unfavorable Mutations for the Synthesis of L-β-Phenylserine

The active site positions, which are located in the aldehyde substrate binding pocket (Y32, F89, K224), as well as positions important for the cofactor binding (G60, N142, A170, R171, R231) or participate in the C_β_-OH protonation (H85, Y87, H128) reduce the conversion in the aldol addition reaction catalyzed by LTAaj (Figure 5, Table 1). The strongest negative impact on the aldol addition of benzaldehyde **1a** to glycine **2a** was observed for the mutations at residues responsible for the binding and fixation of the PLP-cofactor in the active site. For example, G60C perturbs the hydrogen bonding with the phosphate group of PLP and reduces the retro-aldol activity 10-fold, but gives only 5% conversion in the synthesis of **3a**. The large cavity on the *re*-face of the PLP-ring is beneficial for accommodating large aliphatic and aromatic aldehydes, whereas the introduction of the polar residues (e.g., Thr, Ser, or Asn) in positions 32, 89, and 224 results in the decrease of conversion. The positions Y87 and H128 are responsible for the protonation of C_β_-OH group, as was shown previously (Qin et al., 2014). Their mutation led to significantly decreased conversions in the aldol synthesis of **3a**, whereas for the retro-aldol cleavage the effect was not significant.

### Favorable Mutations for the Synthesis of L-α-Alkyl-β-Phenylserines

**Figure 8:**
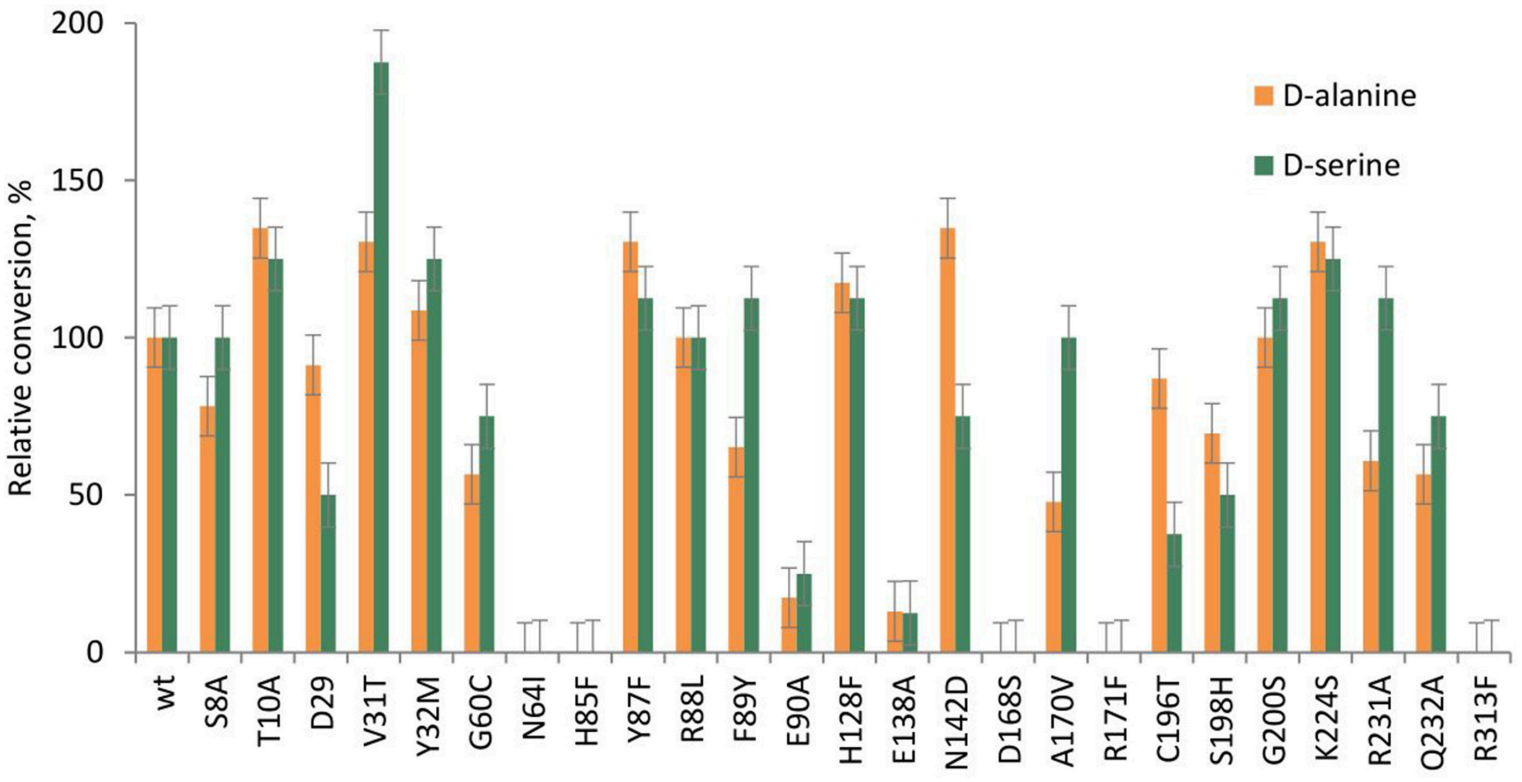
Relative conversion in the aldol addition of benzaldehyde to D-alanine **2b** and D-serine **2c**, catalyzed by LTAaj mutants.

Besides the natural donor glycine **2a**, wild type LTAaj accepts also D-alanine **2b**, and D-serine **2c** in the aldol addition reaction with aldehydes to produce highly valuable L-α-quaternary-α-amino acids (Fesko et al., 2010). In order to improve the performance of biocatalytic transformations, reaction engineering was recently performed and a wide range of L-α-alkyl-β-hydroxy-a-amino acids were produced (Blesl et al., 2018). To further improve the donor specificity and conversions in the reactions, enzyme engineering can be additionally used. Thus, here we have investigated the influence of mutations of active site residues on the aldol synthesis of α-alkyl-β-phenylserines (Figure 8).

**Figure 9:**
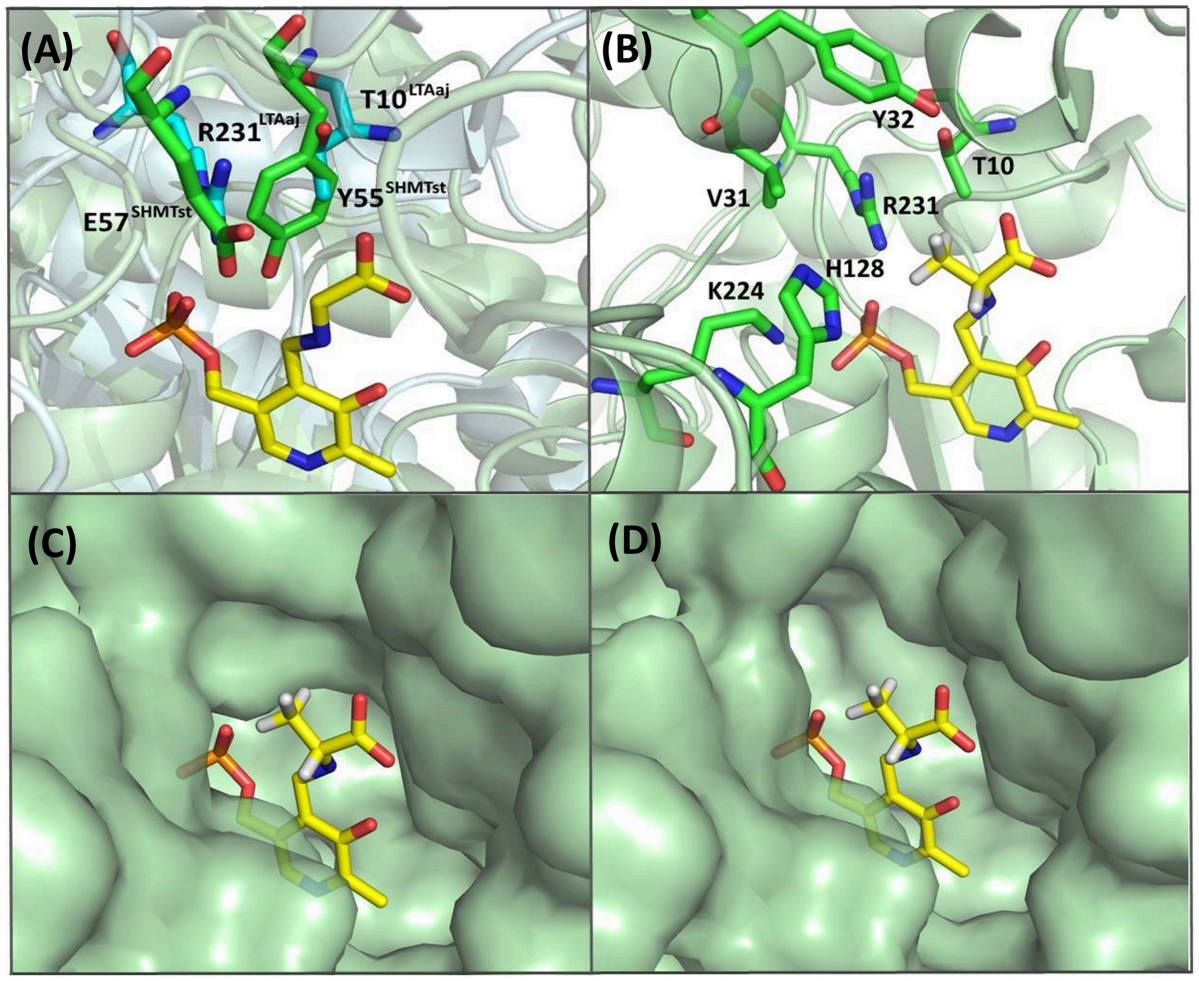
**(A)** Alignment of active site residues of SHMTst (green) and LTAaj (blue); **(B)** LTAaj active site residues, which influence the donor specificity; **(C)** wild type LTAaj active site pocket; **(D)** LTAaj R231A active site pocket.

Recently, serine hydroxy-methyltransferase from *Streptococcus thermophiles* (SHMTst) was engineered to accept D-serine as a donor in the aldol reaction with aldehydes (Hernandez et al., 2015). The mutation Y55T was found to be beneficial to introduce the novel donor specificity presumably due to the generated space which allows to accommodate the (hydroxy)methyl group of the D-serine donor. Additionally, the hydroxy-group of T55 stabilizes the donor in the proper orientation for C_α_-H proton abstraction via hydrogen bonding. In LTAaj T10 occupies the same space as Y55 in SHMTst (Figure 9A). Indeed, the T10A LTAaj mutant increased the conversion by 1.5 fold in the aldol addition of **1a** to **2b** to produce **3b** with up to 70% conversion (Figure 8, Table 1). Besides T10, we have found positions D29, V31, Y32, H128, R231, and K224, which interact with the side-chain of an amino-acid donor, to have an impact on the donor specificity. The mutations V31T, Y32M, and K224S increased the substrate binding pocket to accommodate the side chain of a donor and thus improved the conversion in the aldol reaction with **2b** and **2c** (Figure 9B).

**Figure 10:**
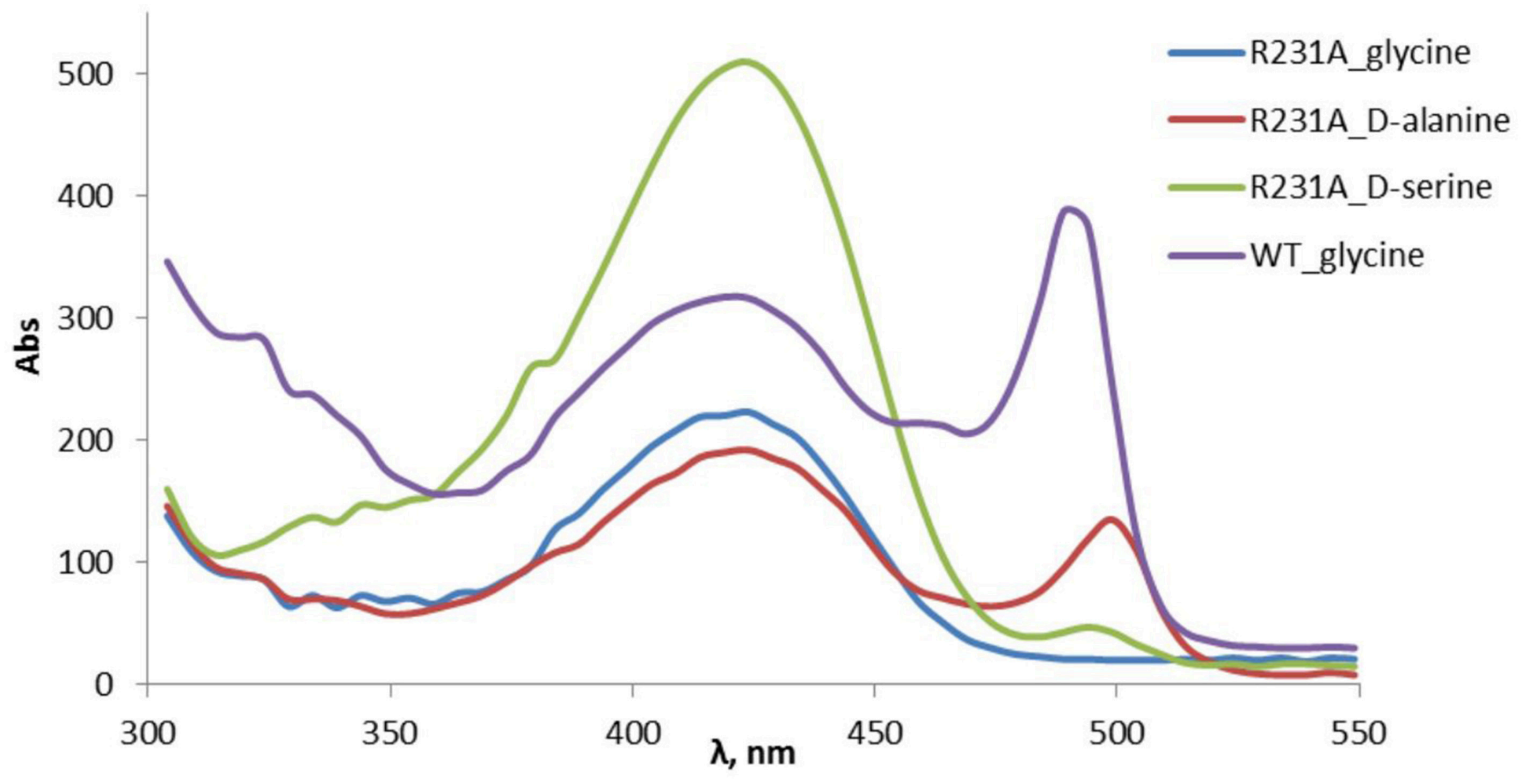
Formation of PLP-quinonoid complex (λ_max_ ≈ 495 nm) with the amino acid donor substrates in the wild type and R231A mutant of LTAaj.

The change of arginine at position 231 to a smaller residue would create even more space and one would expect increased donor specificity for R231X mutants (Figures 9C,D). We performed the site-saturation mutagenesis at R231 in order to find an appropriate residue for this position and tested the variant for the synthesis of **3b**, **3c**, and **3d**. The substitution to the positively charged amino acids (e.g., Lys, Gln) did not significantly influence the conversion. However, small aliphatic residues or residues bearing a negative charge decreased the conversion despite enlarged space upon mutation in comparison to the wild type (Table 2). This might be due to the important role of the residue R231 in the catalytic mechanism. It is a conserved residue among L-*allo* specific threonine aldolases, which participates in the hydrogen bonding with the phosphate group of PLP, as well as maintains the pKa of Schiff-base forming lysine K199 and acts as the general acid/base in the external aldimine formation (Fesko et al., 2018). Moreover, the spectrophotometric analysis showed that R231A mutation hinders the formation of the quinonoid complex with glycine (Figure 10) and, consequently, reduces the natural retro-aldol activity toward L-β-phenylserine by 10 fold. This confirms that R231 is an important catalytic residue, which participates in maintaining the hydrogen network in the active site for donor stabilization.

**Table 2:** Conversions and the diastereomeric ratio (*syn*:*anti*) after 24 h in the aldol addition of benzaldehyde (**1a**) to glycine (**2a**), D-alanine (**2b**), D-serine (**2c**), and D-aminobutanoic acid (**2d**) catalyzed by R231X LTAaj mutants.

**Position**	**Mutants**	**Aldol^a^**							
		**2a**		**2b**		**2c**		**2d**	
		**Conv.%**	**d.r**.	**Conv.%**	**d.r**.	**Conv.%**	**d.r**.	**Conv.%**	**d.r**.
R231	A	20	70:30	14	60:40	9	45:55	< 1	n.d.
	K	15	60:40	18	60:40	7	55:45	n.d.	–
	G	14	65:35	17	60:40	9	55:45	2	20:80
	F	16	70:30	10	65:35	8	55:45	n.d.	–
	T	14	65:35	14	63:37	9	55:45	n.d.	–
	C	12	60:40	21	63:37	7	50:50	n.d.	–
	H	14	60:40	23	60:40	10	50:50	3	50:50
	D	20	70:30	5	60:40	8	42:58	n.d.	–
	V	16	60:40	19	55:45			3	n.d.
	Q	20	60:40	24	55:45	5	45:55	3	n.d.
	M	18	60:40	19	55:45			3	n.d.

a *Reaction conditions: 0.5 U (for **2a**) or 5U (for **2b–d**) of enzyme, 50 mM of **1a**, 500 mM of **2a–c**, 20 mM NaH_2_PO_4_ buffer pH 8.0, 1 mL, 25°C. Products detected by HPLC/LC-MS; e.e._L_>99%*.

*n.d., not determined*.

Interestingly, the quinonoid species were formed in the R231A mutant with non-natural donors, e.g., **2b** and **2c** (Figure 10). This might be due to the divergent mechanism of the fixation of the donors in the active site for C_α_-H abstraction and carbanion stabilization. Indeed, different mutations improve the conversion in the aldol reactions with different donors. Thus, for donor **2b**, the best conversion was obtained with the T10A mutant (Table 1). The conversion with **2c** donor was increased upon mutation to V31T, where T31 might stabilize the donor in a proper orientation via hydrogen bonding with the hydroxy group of serine substrate. For **2d**, the double mutant R231A/Y32M increased the conversion from 0.5 to 8%, presumably due to improved hydrogen stabilization between the side chain of **2d** and M32. Further, H128Y increases the conversion in the aldol reaction with both **2b** and **2c** by providing a hydrophobic environment for the side chain of an amino acid product. Interestingly, H128 participates in the C_β_-OH protonation in the reaction with natural donor glycine **2a** (Qin et al., 2014; Fesko et al., 2018), therefore conversion toward **3a** is reduced for H128F and H128Y mutants (Table 1). In the case of non-natural donors **2b-2d**, the reactions were not affected by the H128F mutation, which indicates that other residue protonate C_β_-O^−^ toward α-quaternary amino acids.

As was shown before, aldehyde **1a** is not optimal substrate for LTA-catalyzed reactions and higher conversions can be obtained with the aromatic substrates bearing electron-withdrawing groups (Fesko et al., 2015; Blesl et al., 2018). Thus, we have applied 3-nitrobenzaldehyde (**1b**) as substrate to test other bulky donors with the mutant variants of LTAaj. Consequently, the conversion with the donor **2d** was improved from 2% for the wild type to 33% for the R231A/Y32M mutant (Table 3). Further, the double mutant accepts also **2f** and **2g** as donors, albeit with low efficiency, which were not accepted by the wild type. The decrease of conversion with an increasing donor's side chain can be enforced due to its interaction with the phosphate group of PLP and destabilization of the overall enzyme-substrate complex. Thus, more rational active site design with simulation studies is beneficial for further engineering of donor specificity of LTAaj. To conclude, in order to get other D-donors accepted, the active site should be designed toward a specific target to enlarge the pocket, respectively, and to fix a donor in the optimal position for C_α_-H deprotonation.

**Table 3:** Conversions in the reaction of 3-nitro-benzaldehyde (**1b**) with D-amino acid donors **2a–g** to produce **4** catalyzed by LTAaj mutants.

**Mutant**	**Acceptor**	**Donor**	conv.,^*^ %	**d.r**.
wt	**1b**	**2a**	>95	80:20
		**2b**	60	65:35
		**2c**	15	40:60
		**2d**	2	50:50
T10A	**1b**	**2a**	>95	80:20
		**2b**	80	68:32
		**2d**	7	70:30
V31T	**1b**	**2d**	26	58:42
N64I	**1b**	**2a**	86	75:25
H85F	**1b**	**2a**	51	65:35
R231Q	**1b**	**2d**	19	65:35
R313F	**1b**	**2a**	>95	70:30
R231A/Y32M	**1b**	**2c**	15	50:50
		**2d**	33	63:38
		**2e**	0	–
		**2f**	2	58:42
		**2g**	1	>95:5

* *Reaction conditions: 0.5 U (for **2a**) or 5U (for **2b–g**) of enzyme, 50 mM of **1b**, 500 mM of **2a–g**, 10%v/v DMSO, 20 mM NaH_2_PO_4_ buffer pH8.0, 1 mL, 25°C. Products detected by HPLC-MS; e.e._L_>99%; d:r = syn:anti*.

### Unfavorable Mutations for the Synthesis of L-α-Alkyl-β-Phenylserines

The conserved residue H85 in LTAaj has an important multifunctional role to (1) coordinate a catalytic water molecule in the active site (di Salvo et al., 2014), (2) to control the electrophilic properties of the PLP ring (Fesko et al., 2018), and (3) to maintain the diastereoselectivity. Although the H85F mutant was active in the aldol addition of **1a** to **2a**, the mutant was inactive in the reaction with non-natural donors. The same is true for other variants, which have an impact on the electrophilic properties of the PLP cofactor (N64I, H85F, E138A, R171T, and R313H)—they were not active in the synthesis of α-alkyl-β-phenylserines **3b-c** (Table 1). This indicates that C_α_-C_β_ bond formation in the case of C_α_-substituted donors occurs with significantly lower rates in comparison to the glycine donor, thus C_α_ carbanion stabilization by the conjugated PLP system is essential for the catalytic activity with non-natural donors. Therefore, a different set of mutations is needed for improving the synthesis of either L-β-phenylserine or L-α-alkyl-β-phenylserines.

## Discussion

L-threonine aldolases are promising catalysts for the environmentally friendly enzymatic synthesis of enantiopure non-natural amino acids. However, the biotechnological application of the wild type aldolases is hampered by the thermodynamic and kinetic limitations leading to low diastereoselectivity and product yield. We performed site-directed mutagenesis of the active site residues of L-threonine aldolase from *Aeromonas jandaei* and the mutants were functionally characterized in order to elucidate the impact of each residue on the catalytic activity in the retro-aldol cleavage reaction as well as for the synthesis of L-β-hydroxy-α-amino acids. We have found, that positions which are important for the carbanion stabilization (e.g., N64, H85, E138, R171, R313) significantly reduce the specific activity in the cleavage reaction, whereas a high synthetic capacity to produce L-β-phenylserine is maintained. Thus, for the efficient retro-aldol cleavage process catalyzed by LTA, C_α_-carbanion has to be stabilized, whereas this is much less important in the aldol process. The suppression of the retro-aldol activity makes the aldol reaction virtually irreversible. Therefore, these conditions affect the kinetics of the reaction improving the diastereoselectivity and increasing the final conversion to the aldol adducts. Consequently, we could remarkably improve the conversion in the aldol addition of benzaldehyde to glycine from 20 to 60% by applying mutants with low retro-aldol activity. Moreover, L-*anti*-β-phenylserine can be obtained as the major isomer under kinetically controlled conditions with H85F and R313F mutants.

This observation is essential for designing screening experiments to discover new efficient LTA variants. The currently applied screening procedures are mainly based on the detection of initial velocities in the cleavage of a product of interest, where the variants with low retro-aldol activity will be overlooked. However, such variants might be beneficial to run the aldol reaction under kinetic control to allow product accumulation with higher yield. Thus, screening techniques where the formation of the product is monitored should be applied to observe the mutants with improved performance in the aldol reaction.

In contrast, in the aldol reactions with non-natural donors, the C_α_-carbanion stabilization is essential. The mutations, which are beneficial for the conversion in the synthesis of L-β-phenylserine, turned out to be deleterious for the aldol synthesis of L-α-alkyl-β-phenylserine. Further, we have found that a hydrophobic environment of the aldehyde binding pocket is important for an efficient progress of the aldol reaction. The T10A, V31T, Y32M, R231A mutants enlarge the active site pocket to accommodate the side chain of a donor and provide a favorable surrounding to stabilize the donor for C_α_-H abstraction, and thus, increase the donor specificity. We have found that due to the high flexibility of the active site of LTAaj, an amino acid donor has to be specifically fixed in the active site to support C_α_-H abstraction. For each donor molecule, different sets of mutations/positions exist, that favor the donor's binding in an appropriate orientation. Thus, substrate binding should be specifically modulated and rationally designed toward the donor of interest. Here we showed the double mutant R231A/Y32M evolves novel donor specificity of LTAaj to accept D-aminobutanoic acid, D-threonine, and D-norvaline. Although the described mutants are still not suitable for the synthetic applications, they can be used as a starting point for further enzyme engineering with respect to α-quaternary-α-amino acids.

In conclusion, the protein engineering and knowledge of the catalytic function of active site residues facilitated the elucidation of novel LTAaj variants with increased productivity for the synthesis of L-β-phenylserine and L-α-alkyl-β-phenylserine. LTAaj accepts a wide range of aldehyde substrates and the conversion in the aldol reaction depends very much on the nature of the aldehyde substrate used. Thus, upon application of an improved variant with a good electrophile substrate such as 3-nitro-benzaldehyde conversion up to 100% can be reached in the aldol reactions catalyzed by LTAaj.

## Data Availability

All datasets generated for this study are included in the manuscript and/or the Supplementary Files.

## Author Contributions

The author confirms being the sole contributor of this work and has approved it for publication.

### Conflict of Interest Statement

The author declares that the research was conducted in the absence of any commercial or financial relationships that could be construed as a potential conflict of interest.
