# Supplementary material for: Comparison of L-Threonine Aldolase Variants in the Aldol and Retro-Aldol Reactions
Source: Front Bioeng Biotechnol. 2019 May 28;7:119. doi: 10.3389/fbioe.2019.00119 (PMC6546723; doi:10.3389/fbioe.2019.00119)
Supplement: Supplementary file 1 [file Data_Sheet_1.PDF]

Supporting information

Comparison of L-Threonine aldolase variants in the aldol and retro-aldol reactions

Kateryna Fesko[1]

[1]Institute of Organic Chemistry, Graz University of Technology, Graz, Austria
kateryna.lypetska@tugraz.at

SI content:

Figure S1. Sequence of the wild-type LTAaj 2

Figure S2. Sequence alignment of the LTAaj used in this study with the template sequence ... 2

Figure S3. Crystal structure alignment of template PDB:3WGB with the LTAaj model.......... 3

MRYIDLRSDTVTQPTDAMRQAMLHAEVGDDVYGEDPSVNVLEAYGAKLLGKQAALFVPSGTM
SNLLAVMSHCQRGEGAILGAGAHIYRFEAQGSAVLGSVALQPLPMQRDGTLAFDEIKAALAPDD
AHFVQTRLICLENTHNGKVLPLSYLQEMGAFVAKHGLKLHLDGARLFNAAVASDTPAEAIAAPFD
SISICLSKGLGAPVGSLLVGDRDFIARARRLRKMVGGGMRQAGMLAQAGLFALEQHVARLADDH
RRAKRLAEGLAALPGIGLDLSLVQSNMVFLRLAKGEPAQLLAFMKERGILFSGYGELRLVTHLQIND
DDIEEVIDAFTEYLGA

Figure S1. Protein sequence analysis of the wild-type L-threonine aldolase from *Aeromonas jandaei* (LTAaj) used in this study.

```
3WGB_A|PDBID|CHAIN|SEQUENCE      MRYIDLRSDTVTQPTDAMRQCMLHAEVGDDVYGEDPGVNALEAYGADLLG
LTAaj_study                      MRYIDLRSDTVTQPTDAMRQAMLHAEVGDDVYGEDPSVNVLEAYGAKLLG
                                 ********************.***************.**.******.***

3WGB_A|PDBID|CHAIN|SEQUENCE      KEAALFVPSGTMSNLLAVMSHCQRGEGAVLGSAAHIYRYEAQGSAVLGSV
LTAaj_study                      KQAALFVPSGTMSNLLAVMSHCQRGEGAILGAGAHIYRFEAQGSAVLGSV
                                 *:**************************:**:.*****:***********

3WGB_A|PDBID|CHAIN|SEQUENCE      ALQPVPMQADGSLALADVRAAIAPDDVHFTPTRLVCLENTHNGKVLPLPY
LTAaj_study                      ALQPLPMQRDGTLAFDEIKAALAPDDAHFVQTRLICLENTHNGKVLPLSY
                                 ****:*** **:**: :::**:****.**. ***:*************.*

3WGB_A|PDBID|CHAIN|SEQUENCE      LREMRELVDEHGLQLHLDGARLFNAVVASGHTVRELVAPFDSVSICLSKG
LTAaj_study                      LQEMGAFVAKHGLKLHLDGARLFNAAVASDTPAEAIAAPFDSISICLSKG
                                 *:**  :* :***:***********.***. ... :.*****:*******

3WGB_A|PDBID|CHAIN|SEQUENCE      LGAPVGSLLVGSHAFIARARRLRKMVGGGMRQAGILAQAGLFALQQHVVR
LTAaj_study                      LGAPVGSLLVGDRDFIARARRLRKMVGGGMRQAGMLAQAGLFALEQHVAR
                                 ***********.: ********************:*********:***.*

3WGB_A|PDBID|CHAIN|SEQUENCE      LADDHRRARQLAEGLAALPGIRLDLAQVQTNMVFLQLTSGESAPLLAFMK
LTAaj_study                      LADDHRRAKRLAEGLAALPGIGLDLSLVQSNMVFLRLAKGEPAQLLAFMK
                                 ********::*********** ***: **:*****:*:.**.* ******

3WGB_A|PDBID|CHAIN|SEQUENCE      ARGILFSGYGELRLVTHLQIHDDDIEEVIDAFTEYLGA
LTAaj_study                      ERGILFSGYGELRLVTHLQINDDDIEEVIDAFTEYLGA
                                  *******************:*****************
```

Figure S2. Sequence alignment of the LTAaj used in this study with the template sequence (UniProtKB O07051; PDB: 3WGB)

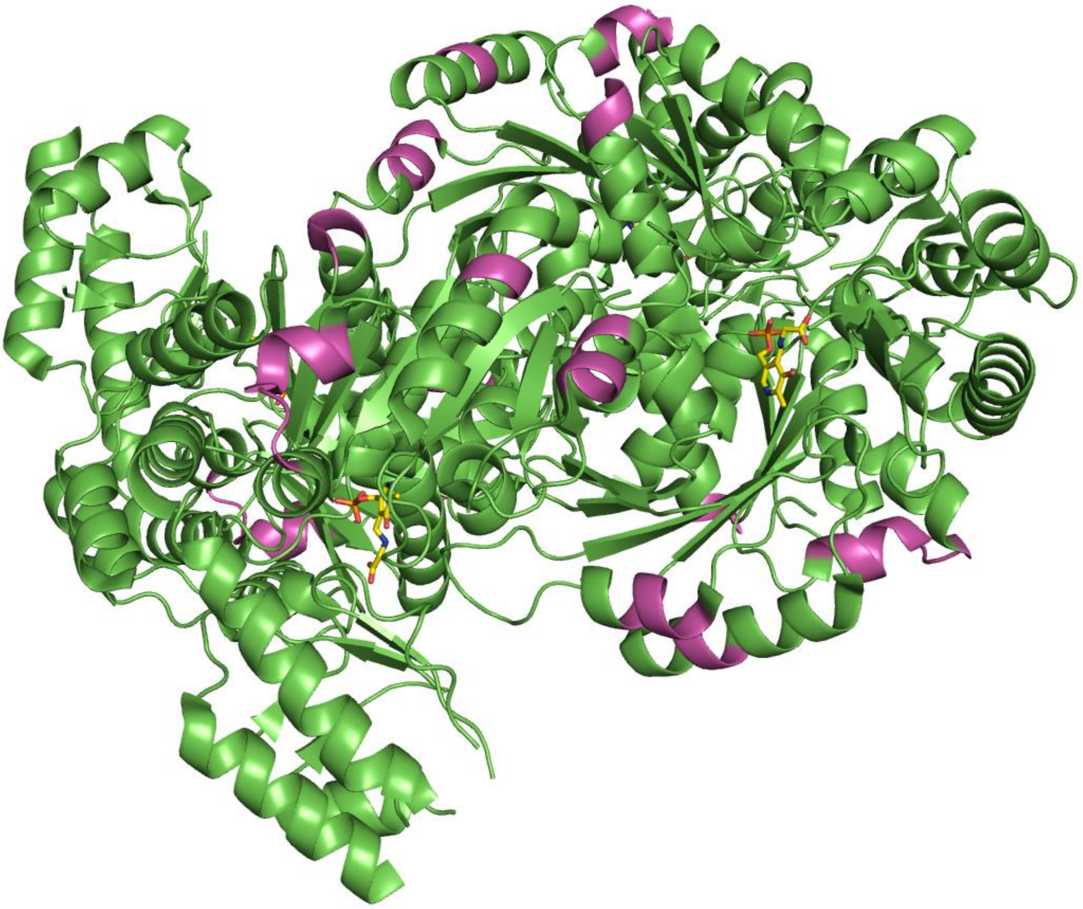

Figure S3. Crystal structure alignment of template PDB:3WGB with the LTAaj model. Pink – not-conserved residues of LTAaj used in study with the template sequence. Not-conserved residues are located in the surface of crystal structure of the protein and do not have significant impact on the active site structure and properties.
